# No potential causal link between HP infection and IBD: A 2way Mendelian randomization study

**DOI:** 10.1097/MD.0000000000037175

**Published:** 2024-02-23

**Authors:** Kaiqi Yang, Yuchen Ding, Jinlong Chen, Xiujing Sun

**Affiliations:** a Department of Gastroenterology, Beijing Friendship Hospital, Capital Medical University, National Clinical Research Center for Digestive Disease, Beijing Digestive Disease Center, Beijing Key Laboratory for Precancerous Lesion of Digestive Disease, Beijing, 100050, China

**Keywords:** causal inference, *Helicobacter pylori* infection, inflammatory bowel disease, Mendelian randomization, null association

## Abstract

Recent epidemiological research suggests a possible negative correlation between *Helicobacter pylori* infection and inflammatory bowel disease (IBD). However, conflicting studies have provided unclear evidence regarding these causal relationships. Therefore, recommending specific prevention and treatment strategies for *H. pylori* infection and IBD is challenging. We used various antibodies (anti-*H. pylori* IgG, VacA, and GroEl) related to *H. pylori* infection as indicators. We acquired relevant genetic variants from public databases within the Genome-wide Association Studies (GWAS) dataset using IBDs tool variables from 2 different GWAS datasets. We thoroughly examined the data and screened for IVs that fulfilled these criteria. Subsequently, Bidirectional Mendelian randomization (MR) was conducted to predict the potential causality between the 2. To ensure the accuracy and robustness of our results, we conducted a series of sensitivity analyses. Based on our comprehensive MR analysis, no potential causal relationship was observed between *H. pylori* infection and IBD. Across various methodologies, including IVW, MR-Egger, and weighted median, our findings showed *P* values > .05. The only exception was observed in the reverse MR analysis using the MR-Egger method, which yielded a *P* value of < .05. However, because the IVW method is considered the most statistically significant method for MR, and its *P* value was > .05, we do not believe that a potential causal relationship exists between them. Our sensitivity analysis did not suggest significant horizontal pleiotropism. Although heterogeneity was detected in the analysis of IBD (IIBDGC source) versus *H. pylori* GroEL antibody levels (MR-Egger, Qp = 0.038; IVW, Qp = 0.043), the results remained reliable because we selected IVW as a random-effects model in our MR analysis method. Based on our MR research, no direct correlation was observed between *H. pylori* infection and IBD risk. This implies that eradicating *H. pylori* may not provide substantial benefits in preventing or treating regional IBD, and vice versa. Nevertheless, the use of *H. pylori* serological index substitution has limitations, and further research using histological diagnosis and additional MR studies is required to comprehensively assess the link between *H. pylori* infection and IBD.

## 1. Introduction

Inflammatory bowel disease (IBD) is a group of idiopathic chronic IBDs, mainly ulcerative colitis (UC), and Crohns disease (CD).^[1]^ IBD seriously affects patients’ quality of life and work capacity, and substantially burdens social development and healthcare systems.^[2]^ IBD affects more than 2.5 million people in Europe and has an increasing incidence in newly industrialized countries in Asia, South America, and the Middle East.^[3]^ Although the pathogenesis of IBD has not yet been fully elucidated, studies have shown that a combination of external environmental factors, intestinal microflora, individual genetic susceptibility, and immune responses influences the pathogenesis of IBD.^[4–6]^

*Helicobacter pylori*, a gram-negative bacterium, is a widely prevalent bacterial infection that has infected nearly 60% of the global population. Moreover, it is an important public health concern requiring attention and effective management.^[7,8]^ A recent meta-analysis showed that the prevalence of *H. pylori* infection in children worldwide is approximately 32%.^[9]^ *H. pylori* has been associated with various diseases, including peptic ulcer (10%), noncardia cancer (1%–3%), and gastric mucosa-associated lymphoid tissue lymphoma (<0.1%).^[10]^ *H. pylori*, which colonizes the digestive system, can not only cause localized inflammatory responses but can influence the patient’s immune system.^[11–14]^ This link has led researchers to explore the potential association between *H. pylori* and the pathogenesis of IBD.

As societies with inadequate living conditions improve sanitation, the prevalence of *H. pylori* infection tends to decrease. Conversely, the incidence of IBD increases in communities that adopt a Western lifestyle.^[15]^ Several other epidemiologic studies have demonstrated similar results,^[16–19]^ suggesting a negative causal link; that is, the widespread eradication of *H. pylori* is detrimental to the prevention and control of IBD. However, contrasting findings have been reported in other studies.^[20–22]^ Additionally, Pokrotnieks et al^[23]^ hypothesized that there was no direct cause-and-effect relationship between *H. pylori* infection and IBD. Instead, the negative correlation established by epidemiological studies may be associated with the transient state of host fucosylated glycans in the gastrointestinal tract. Evidence regarding the relationship between *H. pylori* infection and IBD is based solely on observational studies, which have certain limitations such as confounding factors, reverse causality, and other sources of bias. Currently, a direct causal relationship between *H. pylori* infection and IBD remains unclear. This lack of evidence poses challenges in recommending specific prevention or treatment strategies for *H. pylori* infection and IBD.

Mendelian randomization (MR) is an advanced epidemiological analysis method that facilitates the prediction of causal relationships between factors. MR designs employ genetic variation as an instrumental variable (IV) to capture exposure factors. Genetic variations, typically represented as single-nucleotide polymorphisms (SNPs), are randomly distributed and remain impervious to environmental stimuli and other potential confounding variables. SNPs are genetic variations that occur spontaneously and are entirely independent of environmental factors and other potential confounding variables.^[24]^ With the development of large-scale Genome-wide Association Studies (GWAS) data, MR analyses have become more common, allowing for a rigorous explanation of the causal relationships between complex diseases.

## 2. Materials and methods

### 2.1. Mendelian randomized design

**Figure 1.**
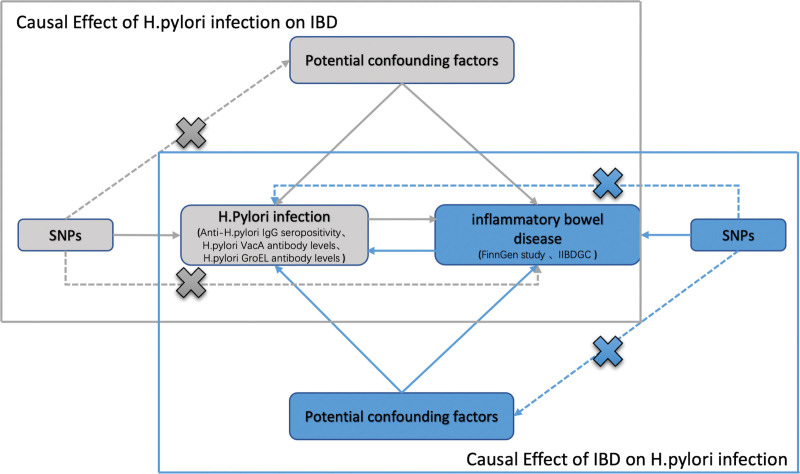
Schematic representation of the bidirectional MR study of the causal relationship between *H. pylori* infection and IBD. SNPs, single-nucleotide polymorphisms. *H. pylori*, *Helicobacter pylori.*

The methodology of this MR study is outlined in detail in Figure 1. Genetic variants were employed as the IV for MR analyses, and the validity of our study relied on 3 fundamental assumptions. The correlation assumption, which asserts that genetic variants are highly correlated with exposure. Second, the independence assumption, which maintains that genetic variants are not influenced by any confounding factors that may mediate the pathway from exposure to outcome. Lastly, the exclusion-restriction assumption, which posits that genetic variants are only expected to impact outcome through exposure.^[25]^

### 2.2. Selection of diagnostic indicators of *H. pylori* infection

The diagnosis of *H. pylori* infection is mainly categorized into invasive (endoscopy-based) and noninvasive methods, each with unique advantages and disadvantages, making the selection of an appropriate diagnostic index for *H. pylori* infection substantial in research. As a noninvasive method, the accuracy of serologic testing is not affected by ulcer bleeding, gastric atrophy, and PPIs or antibiotics, which can lead to false-negative results in other invasive or noninvasive tests.^[26]^ In 1993, a study showed that the sensitivity and specificity of serum anti-*H. pylori* IgG antibody assay for the diagnosis of HP infection were 96.2% and 60.0%, respectively.^[27]^ The lower specificity of anti-*H. pylori* IgG antibodies is mainly due to their persistence in the body even after *H. pylori* eradication. This means that a positive result indicates a current or past infection. In a study conducted among adolescents in 2014, the sensitivity and specificity of IgG antibody testing were 91.2% and 97.4%, respectively.^[28]^ Other *H. pylori*-associated serological markers may provide stronger indications of *H. pylori* infection. Epplein et al identified the 4 serologic antibodies with the highest sensitivities associated with *H. pylori* infection as: the VacA antibody (sensitivity: 100%; 95% CI: 84%–100%), followed by GroEl (sensitivity: 95%; 95% CI: 76%–100%), HcpC (sensitivity: 80%; 95% CI: 57%–82%), and HP1564 (sensitivity: 75%; 95% CI: 53%–59%).^[29]^ VacA is a major virulence factor produced by *H. pylori* that facilitates gastric colonization by influencing human epithelial cells.^[30]^ GroEL, the molecular chaperone of *H. pylori*, is a homolog of heat shock protein (HSP60) in prokaryotes and is an essential pathogenesis-associated antigenic component whose primary function is to facilitate protein transport, complete correct folding, and restore the natural conformation. Previous studies have shown that more than 80% of the *H. pylori*-infected population produces antibodies against GroEL.^[31,32]^ Unfortunately, we did not find any GWAS data related to HcpC and HP564 antibodies. In summary, we used *H. pylori*-related antibody indicators of *H. pylori* infection, including serum anti-*H. pylori* IgG, VacA, and GroEl antibodies.

### 2.3. Description of data sources

This study identified 2 distinct genetic associations for IBD using independent GWAS datasets. The first dataset included 5673 individuals of European descent with IBD and 213119 European controls from the FinnGen database, ensuring an accurate and consistent diagnosis of IBD through electronic medical records. The second IBD-related GWAS dataset comprised 12,882 European patients and 34,652 European controls from the International Inflammatory Bowel Disease Genetics Consortium. Additionally, the GWAS summary statistics for anti-*H. pylori* IgG antibody positivity and *H. pylori* VacA and GroEL antibody levels were obtained from public data in the EBI database, including 8735 European patients. Each GWAS was approved by an appropriate ethics committee. For more detailed information, please see Table 1.

**Table 1:** Details of the studies included in the Mendelian randomization analyses

Phenotype	Consortium	Ethnicity	Sample size	Year	Number of SNPs	Web source
IBD	FinnGen study	European	5673	2021	16,380,466	https://gwas.mrcieu.ac.uk/datasets/finn-b-K11_IBD/
IBD	IIBDGC	European	12,882	2015	12,716,084	https://gwas.mrcieu.ac.uk/datasets/ieu-a-31/
Anti-*H. pylori* IgG seropositivity	UK biobank	European	8735	2020	9170,312	https://gwas.mrcieu.ac.uk/datasets/ebi-a-GCST90006910/
*H. pylori* VacA antibody levels	UK biobank	European	1571	2020	9178,635	https://gwas.mrcieu.ac.uk/datasets/ebi-a-GCST90006916/
*H. pylori* GroEL antibody levels	UK biobank	European	2716	2020	9172,299	https://gwas.mrcieu.ac.uk/datasets/ebi-a-GCST90006913/

IBD = inflammatory bowel disease, IIBDGC = International Inflammatory Bowel Disease Genetics Consortium

### 2.4. Selection of genetic instrumental variable (IV)s for *Helicobacter pylori*-related antibodies

The IVs of the 3 *H. pylori*-associated antibodies were obtained separately by GWAS pooling statistics, and a series of quality control measures were used to screen the IV genotypes that met the MR hypothesis. On 1 hand, achieving genome-wide significance for the screened IVs was necessary. On the other hand, obtaining an adequate number of IVs after screening was crucial to enhance statistical power. Hence, the *P*-value threshold for *H. pylori*-associated antibody IVs was < 5 × 10^−6^. Subsequently, a chain imbalance (LI) with a *P*-value < 5 × 10^−8^ was established using an R2 of < 0.001, a window size of 10,000 kb, and a *P*-value < 5 × 10^−8^ for the linkage disequilibrium (LD) clustering algorithm. To confirm that the affected alleles were of the same type, the exposure and resulting datasets were harmonized. This process involved the removal of SNPs with intermediate allele frequencies and ambiguous SNPs with conflicting alleles. Additionally, the F-statistic for each SNP was calculated using the following formula to assess the strength of the IV:


$$\begin{array}{l} F=\frac{N-K-1}{K}\times \frac{R^{2}}{1-R^{2}} \end{array}$$

Where *N* is the sample size, *K* is the number of SNPs, and R2 represents the proportion of variation explained by the independent variable.^[33]^ The F-statistics for all SNPs were > 10. Therefore, SNPs that have been rigorously screened were used for subsequent analyses.

### 2.5. Selection of genetic instrumental variable (IV)s for IBD

Similarly, we performed GWAS pooling statistics to obtain gene IVs for IBD. First, we set relevant parameters to achieve quality control, including *P* < 5 × 10^−8^ to reach genome-wide significance of the SNPs and established the same chained disequilibrium (LD) clustering algorithm with an R2 < 0.001, a window size of = 10,000 kb, and *P* < 5 × 10, balanced (LD) clustering algorithm, and finally harmonized the exposure and resultant datasets. The same formula was used to calculate the F-statistic for each SNP. The SNPs screened after the above steps were used as IV for subsequent analyses.

### 2.6. Statistical analysis and data visualization

Statistical analyses and data visualization were performed using R programming software, version R4.2.3. About 3 complementary methods, inverse-variance weighted, MR-Egger, and weighted median, were utilized for the MR analysis to ensure robustness. The IVW method is the primary causal estimation method, providing the most accurate results when all selected SNPs are valid IVs.^[34]^ The MR-Egger method utilizes a weighted linear regression to obtain consistent causal estimates, assuming that instrumental strength is independent of the direct effect (InSIDE), even when genetic IVs are not valid. However, this method has low precision and is vulnerable to peripheral genetic variation.^[35]^ The weighted median method is a statistical technique that calculates the weighted median of the Wald ratio estimates and does not require the InSIDE assumption. This method is resistant to horizontal pleiotropic biases. Compared to the MR-Egger method, the weighted median process has a lower type I error and is more effective for causal estimation.^[36]^ These methods are provided by the “TwoSampleMR” R package (Version 0.5.7).^[36]^ Significance was attributed to 2-sided *P* values of < 0.05. The generation of forest plots was accomplished through utilization of the “forestploter” R package, with version 2.0.1 implemented for this purpose.

## 3. Results

### 3.1. The causal effect of *Helicobacter pylori* infection on IBD

**Figure 2.**
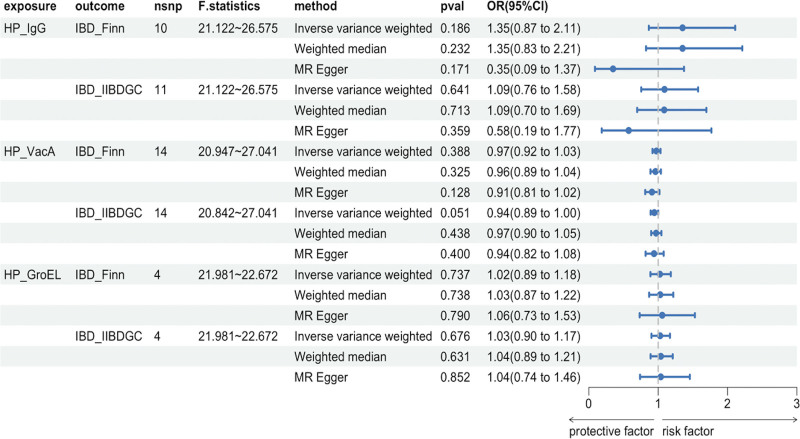
Forest plot of Mendelian randomization analyses. OR, odds ratio. 95%CI, 95% confidence interval. nsnp, number of single-nucleotide polymorphisms. HP_IgG, Anti-*H. pylori* IgG seropositivity. HP_VacA, *H. pylori* VacA antibody levels. HP_GroEL, *H. pylori* GroEL antibody levels. IBD_Finn, GWAS data source of inflammatory bowel disease in the FinnGen database. IBD_IIBDGG, GWAS data source of inflammatory bowel disease from the International Inflammatory Bowel Disease Genetics Consortium.

**Figure 3.**
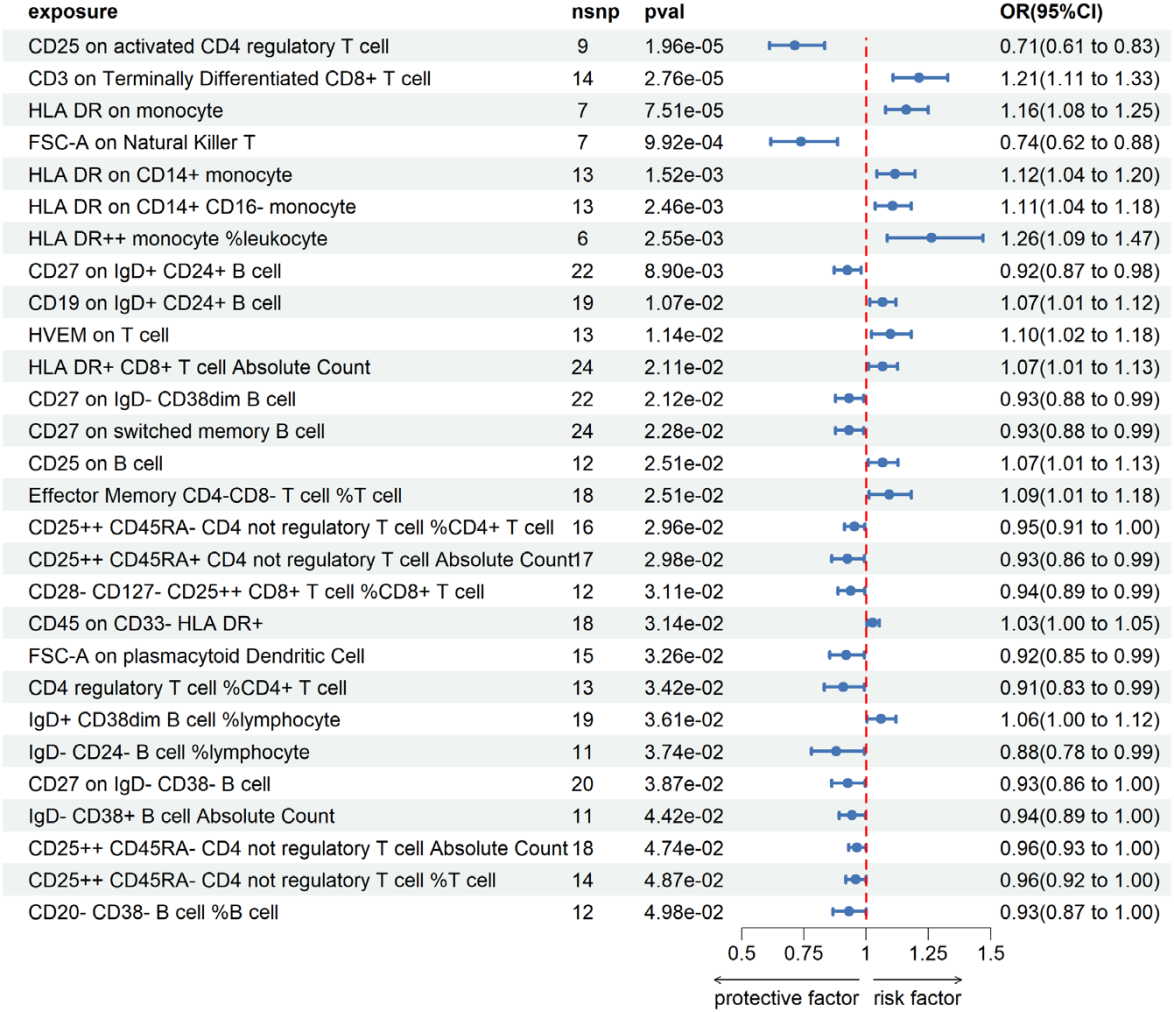
Scatterplot of significant causal relationships between *H. pylori* infection-related antibody markers and IBD. IBD (Finn), GWAS data source of inflammatory bowel disease in the FinnGen database. IBD (IIBDGG), GWAS data source of inflammatory bowel disease from the International Inflammatory Bowel Disease Genetics Consortium.

MR analysis was performed to investigate the causal relationship between *H. pylori* infection and IBD. Various methods including the IVW, MR-Egger, and weighted median were used to validate the results (Fig. 2). All results showed a *P*-value > 0.05. Figure 3 shows the scatter plots used to visualize the data. The findings of the MR analysis showed that *H. pylori* infection does not have a causal effect on IBD.

### 3.2. IBD on *Helicobacter pylori* infection causality

**Figure 4.**
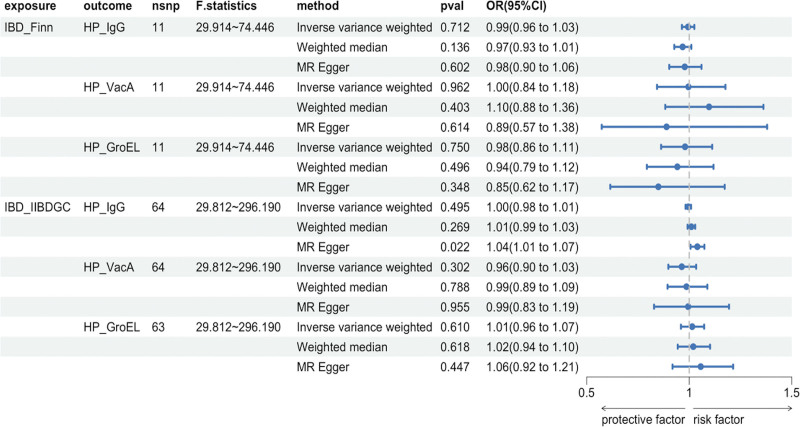
Forest plot of reverse Mendelian randomization analyses. OR, odds ratio. 95%CI, 95% confidence interval. HP_IgG, Anti-*H. pylori* IgG seropositivity. HP_VacA, *H. pylori* VacA antibody levels. HP_GroEL, *H. pylori* GroEL antibody levels. IBD_Finn, GWAS data source of inflammatory bowel disease in the FinnGen database. IBD_IIBDGG, GWAS data source of inflammatory bowel disease from the International Inflammatory Bowel Disease Genetics Consortium.

Furthermore, MR analysis was used to examine the causal relationship between IBD development and *H. pylori* infection. The antibody-mediated cross-reactivity of HPs GroEL, which has a high homology with human HSP60, can contribute to the development of atherosclerosis. Therefore, atherosclerosis-associated Coronary artery disease may be a potential confounding factor. To exclude the effects of horizontal pleiotropy, we excluded rs56062135, which can cause coronary artery disease, from the 2-sample analysis of GroEL antibodies against IBD (IIBDGC source) and HP.^[37]^ Figure 4 shows the results of this study. We utilized the MR-Egger method to analyze the IgG antibody-positive samples for IBD (IIBDGC source) compared to anti-HP. The IVW method indicated a *P*-value of not more than 0.05, implying that no statistically significant causal relationship existed between the 2. The weighted median method yielded results that were consistent with those of the IVW method. However, MR-Egger resulted in a *P*-value < 0.05, with the beta direction being the same for several methods. As the IVW method is the most statistically significant method for MR, we do not believe that a potential causal relationship exists between the 2. The analysis did not establish causality between IBD and *H. pylori* infection.

### 3.3. Sensitivity analysis

We first analyzed all data for heterogeneity, mainly using IVW methods and MR-Egger regression, quantified using Cochrans Q-statistics. Although heterogeneity was detected in the analysis of IBD (IIBDGC source) versus *H. pylori* GroEL antibody levels (MR-Egger, Qp = 0.038; IVW, Qp = 0.043), because we chose the IVW of the random-effects model for the MR analysis method, the results of the study are still reliable.^[38]^ We utilized the MR-PRESSO method to identify the horizontal polytropy. This method can detect horizontal polytropy anisotropy, correct horizontal polytropy by removing outliers, and determine whether there is a significant change in the causal effects before and after removing outliers.^[39]^ The results for MR-PRESSO are shown in Table 2, with *P* < .05, which suggests substantial pleiotropy.

**Table 2:** Results of sensitivity analysis for bidirectional Mendelian randomization

Exposure	Outcome	MR-Egger			IVW			MR-PRESSO
		Q	Q_df	Q_pval	Q	Q_df	Q_pval	pval
HP_IgG	IBD_Finn	9.216	8	0.324	13.912	9	0.126	0.162
	IBD_IIBDGC	13.443	9	0.144	15.525	10	0.114	0.144
HP_VacA	IBD_Finn	11.915	12	0.453	13.808	13	0.387	0.410
	IBD_IIBDGC	4.594	12	0.970	4.599	13	0.983	0.986
HP_GroEL	IBD_Finn	0.359	2	0.836	0.394	3	0.942	0.979
	IBD_IIBDGC	1.900	2	0.387	1.903	3	0.593	0.681
IBD_Finn	HP_IgG	9.827	9	0.365	10.035	10	0.437	0.288
	HP_VacA	10.420	9	0.318	10.769	10	0.376	0.457
	HP_GroEL	9.924	9	0.357	10.903	10	0.365	0.421
IBD_IIBDGC	HP_IgG	70.143	62	0.223	79.426	63	0.079	0.078
	HP_VacA	76.858	62	0.097	77.027	63	0.110	0.102
	HP_GroEL	81.901	61	0.038	82.408	62	0.043	0.125

IVW, Inverse-variance weighted

### 3.4. Meta-merging after MR analysis

In summary, we analyzed the results of different sources of IBD-related GWAS data separately. To further validate the robustness of the results, we performed a meta-analysis of the IVW method results to explore whether the data before and after merging remained consistent. The merged data supported the absence of a causal relationship between *H. pylori* infection and IBD, as shown in Table 3.

**Table 3:** Pooled results were analyzed using meta-analysis. Q_pval, the pval of Cochrans Q-statistics measure

Exposure	Outcome	OR (95%CI)	Pval	Q_pval
HP_IgG	IBD	1.190 (0.896–1.579)	0.230	0.468
HP_VacA	IBD	0.975 (0.928–1.024)	0.309	0.121
HP_GroEL	IBD	1.016 (0.917–1.125)	0.763	0.769
IBD	HP_IgG	0.995 (0.982–1.009)	0.476	0.807
IBD	HP_VacA	0.989 (0.962–1.017)	0.454	0.424
IBD	HP_GroEL	1.013 (0.961–1.068)	0.640	0.836

## 4. Discussion

Evidence from previous studies on the causal relationship between HP infection and IBD has been inconsistent. *H. pylori* belongs to the genus Helicobacter and is usually observed on the surface epithelium of the stomach; however, *H. pylori* DNA has been observed in the colon.^[40]^ *H. pylori* has the potential to induce the production of inflammatory factors in the gastric mucosa. The localized expression of these factors may elicit an immune response at extra-gastric sites, suggesting that HP infection could be implicated in certain autoimmune diseases.^[41–43]^ In IBD, dysregulation of the immune response to commensal bacteria is a crucial pathogenic mechanism. The pathogenesis of IBD, particularly CD, is associated with the TH1 immune response and secretion of pro-inflammatory cytokines.^[44]^ A few patients diagnosed with IBD may suffer from concomitant autoimmune diseases and autoimmune extraintestinal manifestations. Previous studies have indicated that the risk of autoimmune diseases is higher in patients with IBD than in those without.^[45]^ The immunobiological similarity between IBD and *H. pylori* infection provides the context for a possible causal relationship.

Various observational studies have linked Helicobacter spp., including *H. pylori*, to the development of IBD. About 1 study identified the presence of *H. pylori* in the intestinal mucosa of patients with UC-like CD and UC. These findings suggest an association between *H. pylori* infection and IBD pathogenesis. Further research is warranted to elucidate the mechanisms underlying this relationship and determine the clinical implications of these observations.^[46,47]^ In another study, *H. pylori* was detected in fecal specimens of most children with CD.^[48]^ Similarly, Laharie et al reported that CD was significantly associated with *Helicobacter pullorum* or *Helicobacter canadensis* infection in intestinal biopsies (OR = 2.58; 95% CI: 1.04–6.67).^[49]^ However, other studies yielded contradictory results. In an analysis of intestinal mucosal specimens from patients with IBD, 2 Helicobacter genus-specific PCR assays, 2 *H. pylori*-specific assays, and a PCR assay designed to amplify fragments of H. heilmannii-like organisms did not reveal any Helicobacter species, which may suggest that Helicobacter is not involved in IBD. A few experts have suggested that H. heilmannii does not play a role in the pathogenesis of IBD.^[50]^ As per the findings of another analogous research study, the presence of Helicobacter species was not identified in the colon biopsies of patients diagnosed with IBD.^[51]^

Moreover, epidemiological evidence indicates a potential inverse correlation between the prevalence of IBD and *H. pylori* infection. Regions with a low occurrence of *H. pylori* infection have experienced a gradual increase in the incidence of IBD following the widespread implementation of treatment plans aimed at eradicating *H. pylori*.^[52]^ According to a recent meta-analysis, a consistent negative correlation appears to exist between gastric *H. pylori* infection and IBD, with a statistically significant *P*-value < 1e^−10^. This correlation appeared to be more pronounced in patients with CD and IBDU (*P*-value = 0.38 and 0.43) than in patients with UC (*P*-value = 0.53) and (*P* < 1e^−10^).^[53]^ Several other studies have yielded similar results,^[54–58]^ suggesting that *H. pylori* infection may play a protective role against IBD. Studies have suggested that *H. pylori* infection may help in the treatment of IBD, while others disagree. Biopsies revealed no difference in Helicobacter detection between patients with IBD and those with other diseases.^[59]^ In another PCR sequencing study in a Canadian population, no significant difference was observed in the proportion of Helicobacter DNA detected in ileal or colonic samples between patients with IBD and controls. All positive models yielded helicobacter DNA sequences identical to *H. pylori* 16S rDNA, which was similar to *Helicobacter. cinaedi* (AF348745), *Helicobacter fennelliae* (AF348746), *Helicobacter heilmannii* (AF058768), *Helicobacter bilis* (AF047843), and *Helicobacter hepaticus* (AF302103) all differed in 2.5%, 2.5%, 3.6%, 3.9%, and 4.2% of the corresponding sequences, respectively.^[60]^ This finding suggests that no correlation exists between *H. pylori* infection and IBD.

The controversy surrounding whether *H. pylori* infection causes IBD arises primarily because of the limitations of observational studies. These studies did not include randomization, prospective analysis, or blinding to account for confounding factors. In contrast, MR selects SNPs as IVs that remain unaffected by environmental and other exposure factors owing to their random distribution.^[24]^ Therefore, we chose MR to analyze *H. pylori* infection and IBD, which could strictly explain the causal relationship between them.

We used various MR techniques to investigate the causal relationship between *H. pylori* infection and IBD. However, our extensive bidirectional MR analysis did not provide evidence of a genetically predicted causal association between these 2 conditions.

Despite our efforts, this study has limitations that are worth noting. *H. pylori*-related antibody indicators were used to assess *H. pylori* infection. However, these indicators have certain drawbacks that could potentially skew our results, even though we carefully selected several indicators with sensitivity levels exceeding 90%. Furthermore, we acknowledge that there may have been an overlap between participants in the exposure and outcome samples. This overlap may have impacted the accuracy of the MR analysis. However, we attempted to minimize any potential bias by utilizing IVs with strong effects, all of which boasted F-statistics in excess of 10. Our dataset included only European populations. While this choice helped reduce population stratification bias, it is possible that our findings may not be generalizable to other populations.

## 5. Conclusion

In our study using MR, no direct link was observed between *H. pylori* infection and the risk of IBD. This suggests that there may not be significant benefits in eradicating *H. pylori* to prevent or treat regional IBD, and vice versa. However, the limitations of *H. pylori* serologic index substitution indicate that additional GWAS research based on histologic diagnosis, and further MR studies, are necessary to fully evaluate the relationship between *H. pylori* infection and IBD.

## Author Contributions

**Data curation:** Kaiqi Yang.

**Methodology:** Kaiqi Yang.

**Software:** Kaiqi Yang.

**Writing—original draft:** Kaiqi Yang.

**Visualization:** Yuchen Ding.

**Resources:** Jinlong Chen.

**Writing—review & editing:** Xiujing Sun.
